# Integrated Analytical System for Comprehensive Two-Dimensional Enantio-Gas Chromatography and Low-Pressure Gas Chromatography Mass Spectrometry Utilizing a Switching Valve: Design and Optimization

**DOI:** 10.1021/acs.analchem.5c05852

**Published:** 2025-11-12

**Authors:** Giorgia Rinaldi, Antonio Ferracane, Mariosimone Zoccali, Luigi Mondello

**Affiliations:** † Messina Institute of Technology c/o Department of ChemicalBiological, Pharmaceutical and Environmental Sciences, former Veterinary School, University of Messina, Viale G. Palatucci snc, 98168 Messina, Italy; ‡ Department of Mathematical and Computer Science, Physical Sciences and Earth Sciences, 18980 University of Messina, Viale F. Stagno d’Alcontres 31, 98166 Messina, Italy; § Chromaleont s.r.l., c/o Messina Institute of Technology c/o Department of Chemical, Biological, Pharmaceutical and Environmental Sciences, 550300 University of Messina, Viale G. Palatucci snc, 98168 Messina, Italy

## Abstract

An analytical system was developed to perform both comprehensive two-dimensional enantio-gas chromatography mass spectrometry with the second-dimension column working under low-pressure conditions (eGC×LP-GC-QMS) and low-pressure gas chromatography mass spectrometry (LP-GC-QMS) analysis, without any changes to the instrumental setup. This is achieved using a switching valve, which also allows for back-flushing of the first-dimension column, thereby generating a flexible and powerful analytical platform. The eGC×LP-GC-QMS configuration can provide both targeted and untargeted information in a single run. In this research, it is employed for the analysis of 56 pesticides (without considering isomers), with the added capability of the backflush mode, effectively removing high-boiling compounds extracted from the matrix and improving sample throughput and system robustness, without major concerns regarding sample preparation. The first-dimension is a chiral column, which enables the separation of seven enantiomers: acephate, benoxacor, chlorflurecol, dibrom, fipronil, methamidophos, and propetamphos. The second-dimension is a wide-bore column (5 m × 0.53 mm I.D. × 0.53 μm *d*_*f*_) operating under vacuum conditions. Thanks to the use of a switching valve, it is possible to directly perform LP-GC-QMS analysis, enabling the investigation of a broader range of compounds in terms of boiling point. The operability range of volatile analytes was evaluated for both configurations by injecting different alkane mixtures: C_7_–C_30_ for the eGC×LP-GC-QMS configuration and C_7_–C_40_ for the LP-GC-QMS configuration.

## Graphical abstract

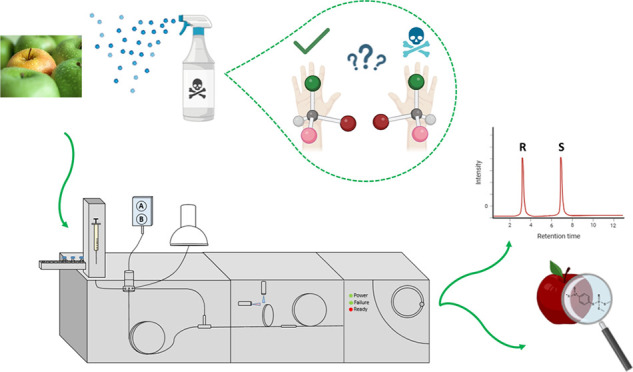

## Introduction

Technological advancements in the analytical field have led to the development of increasingly sophisticated instruments for the detailed analysis of complex samples. Comprehensive two-dimensional gas chromatography (GC×GC) is one such technique, particularly valuable for analyzing volatile and semivolatile compounds.^1,2^

GC×GC is widely employed across various fields, and its application in chiral analysis has also been explored. The study of chiral compounds finds wide application in various fields of chemistry.^3^ For example, in the case of food chemistry, chiral molecules can have different tastes and aromas, different bioavailability and absorption, and can also be used to study the naturalness of flavorings.^4,5^

Studies have demonstrated GC×GC advantages over heart-cutting enantioselective multidimensional gas chromatography, a well-established technique for the targeted analysis of specific chiral compounds.^6^ As shown by Galletta et al., GC×GC can be used for both targeted separation of chiral compounds and untargeted analysis within a single run. Specifically, the study focuses on the targeted analysis of chiral lactones and an untargeted screening of volatile compounds in Marsala wine samples. The volatility range of the analyzed compounds is fully compatible with the temperature limits of chiral stationary phases, which generally do not exceed 230–250 °C.^7^ However, the maximum operating temperature of chiral stationary phases poses a limitation on their applicability. For instance, analyzing chiral pesticides in food samples is particularly challenging due to the coextracted matrix introduced during sample preparation, which may have limited volatility.^8,9^ Considering the significant differences between enantiomers in terms of activity, toxicity and biotransformation, a solution for the enantio-separation is crucial.^10^

Consequently, to overcome this issue and to enhance sample throughput and system robustness while minimizing carryover, the backflush approach was proposed.^11–13^ This technique is commonly used in both mono- and multidimensional systems, typically requiring an additional injector or pressure control.^11,12^ Specifically in the GC×GC field an approach to isolate the first dimension (^1^D) column and backflush the inlet after the injection was proposed by Edwards and Gorécki in 2015.^13^

In the study proposed herein, a switching valve with appropriate connections was used to switch from enantio-GC×GC single quadrupole mass spectrometry, with the second-dimension (^2^D) column working under low-pressure conditions (eGC×LP-GC-QMS), to backflush mode without the need for additional injectors or flow controls. Furthermore, the developed system allows analyses to be conducted under low-pressure gas chromatography mass spectrometry (LP-GC-QMS) conditions without any instrumental modifications, thereby increasing flexibility and productivity.^14,15^

This approach is made possible by using a wide-bore column, ^2^D, placed in a separate oven to overcome the temperature limitations of the first analytical dimension. The advantages of the LP-GC-QMS technique are well-known and well documented, including faster analytical separations thereby increasing productivity while reducing energy consumption and costs.^16^ Compared to narrower columns, wide-bore columns enhance sample capacity, improve robustness, and minimize maintenance requirements.^16^ LP-GC-QMS also produces taller and sharper peaks, lowering limits of quantification (LOQs). Additionally, it can be implemented on most GC instruments using commercially available components, making it accessible to experienced chromatographers.

The use of a wide-bore column as ^2^D was previously employed, for example, by Tranchida et al. in 2014, who proposed low-pressure condition in the ^2^D using a flow modulator interface, and by Corbally et al. in 2023, who used a cryogenic modulator.^17,18^

In this study, the technique was applied to the analysis of pesticides extracted from an apple sample as a proof of concept, expanding the range of analyzable compounds compared to the eGC×LP-GC-QMS technique. This is achieved without requiring instrumental modifications, only a simple valve position change, thus enhancing the analytical system’s productivity. With the developed system it is therefore possible to obtain both a detailed characterization of chiral compounds in targeted and untargeted mode, and a rapid targeted analysis of pesticides with a wider range of boiling points, with also the possibility of back-flushing the chiral ^1^D column. The design and optimization of this system are discussed in detail, highlighting both its strengths and limitations.

## Experimental Section

### Reagents and Chemicals

Pesticide standards (purity ⩾98%), ethyl acetate (EtOAc, grade ≥99.9%), acetonitrile (ACN, grade ≥99.9%), acetone (grade ≥99.9%), triphenyl phosphate (TPP, used as internal standard), C_7_–C_30_ and C_7_–C_40_ saturated alkanes standards mixtures, magnesium sulfate, and sodium chloride were obtained from Merck Life Science (Merck KGaA, Darmstadt, Germany). Stock solutions (10,000 mg L^–1^) were prepared for each individual pesticide in acetone, and stored at 4 °C. The internal standard, TPP, was prepared at the same concentration in ACN, and also stored at 4 °C. An intermediate multianalyte standard solution, containing 56 different pesticides, was prepared at a concentration of 30 mg L^–1^ and stored at 4 °C until use.

### Sample Preparation

Sampling was performed according to the guidelines established by the European standard procedure [EN 15662, 2018].^19^ A portion of the apple, including peel and seeds, was homogenized using a digital homogenizer Ultra-Turrax T 25 (Janke & Kunkel GmbH Co., IKA Labortechnik, Wilmington, NC, USA). A subset of 1 g was spiked with 10 μL of IS solution (50 mg L^–1^) and different concentrations of the pesticide mixture. Extraction was carried out with 1 mL of EtOAc and 500 mg of magnesium sulfate and sodium chloride. The sample was vortexed for 1 min using a Fisher Scientific Digital Vortex Mixture (Fisher Scientific, Pittsburgh, PA, USA) and centrifuged at 4500 rpm for 10 min. Finally, the supernatant was collected, transferred to a vial and concentrated to a final volume of 200 μL under a gentle steam of nitrogen.

### Chromatographic Setup and Conditions

The proposed setup included a dual-oven GC-2010 plus gas chromatography system (Shimadzu Corporation, Kyoto, Japan), a Zoex II cryogenic modulator (a device that traps and focuses analyte bands at very low temperatures between the ^1^D and ^2^D, enabling sharp reinjection and high-resolution separation, in this specific case which operates without the use of liquid nitrogen^1^) installed into the second oven, a single quadrupole mass spectrometer (QP2010, Shimadzu), and an AOC-20i autosampler. The first GC oven was equipped with a split/splitless injector (operating at 230 or 300 °C for the eGC×LP-GC-QMS and LP-GC-QMS, respectively) and a focus liner with a volume of 810 μL.

A 6-port, 2-position high-temperature sampling/switching valve (Valco Instruments Co. Inc., Schenkon, Switzerland) was installed in the first oven and used to switch between the eGC×LP-GC-QMS and the LP-GC-QMS configuration. A scheme of the system is reported in Figure 1. Specifically, port 1 of the valve was connected to the GC injector by using an uncoated capillary column of the following dimensions: 0.34 m × 0.2 mm I.D. The port 2 was connected to the ^1^D chiral column, a MEGA DEX-DET beta (20 m × 0.18 mm I.D. × 0.18 μm *d*_*f*_) (MEGA, Milano, Italy). The outlet of the column was connected to an MXT Y-union (Restek Corporation, Bellefonte, PA, USA). Port 3 was connected to an uncoated capillary column of the following dimensions 0.4 m × 0.05 mm I.D., used as waste during the backflush of the ^1^D chiral column. Ports 4 and 5 were plugged, and port 6 was connected by using an uncoated capillary column of the following dimensions 0.34 m × 0.2 mm I.D., to the Y-union. A nonpolar column, namely, Equity-5 [bonded phase; poly­(5% diphenyl/95% dimethylsiloxane)] (5 m × 0.53 mm I.D. × 0.53 μm *d*_*f*_) (Merck Life Science), was connected to the third port of the Y-union by using an uncoated column of the following dimensions 1.5 m × 0.1 mm I.D. and a SilTite μ-union (Trajan Scientific and Medical, Trajan Scientific Australia Pty Ltd.). Modulation was carried out every 5 s by using a loop-type modulator. The duration of the hot pulse (280 °C) was 300 ms.

**Figure 1:**
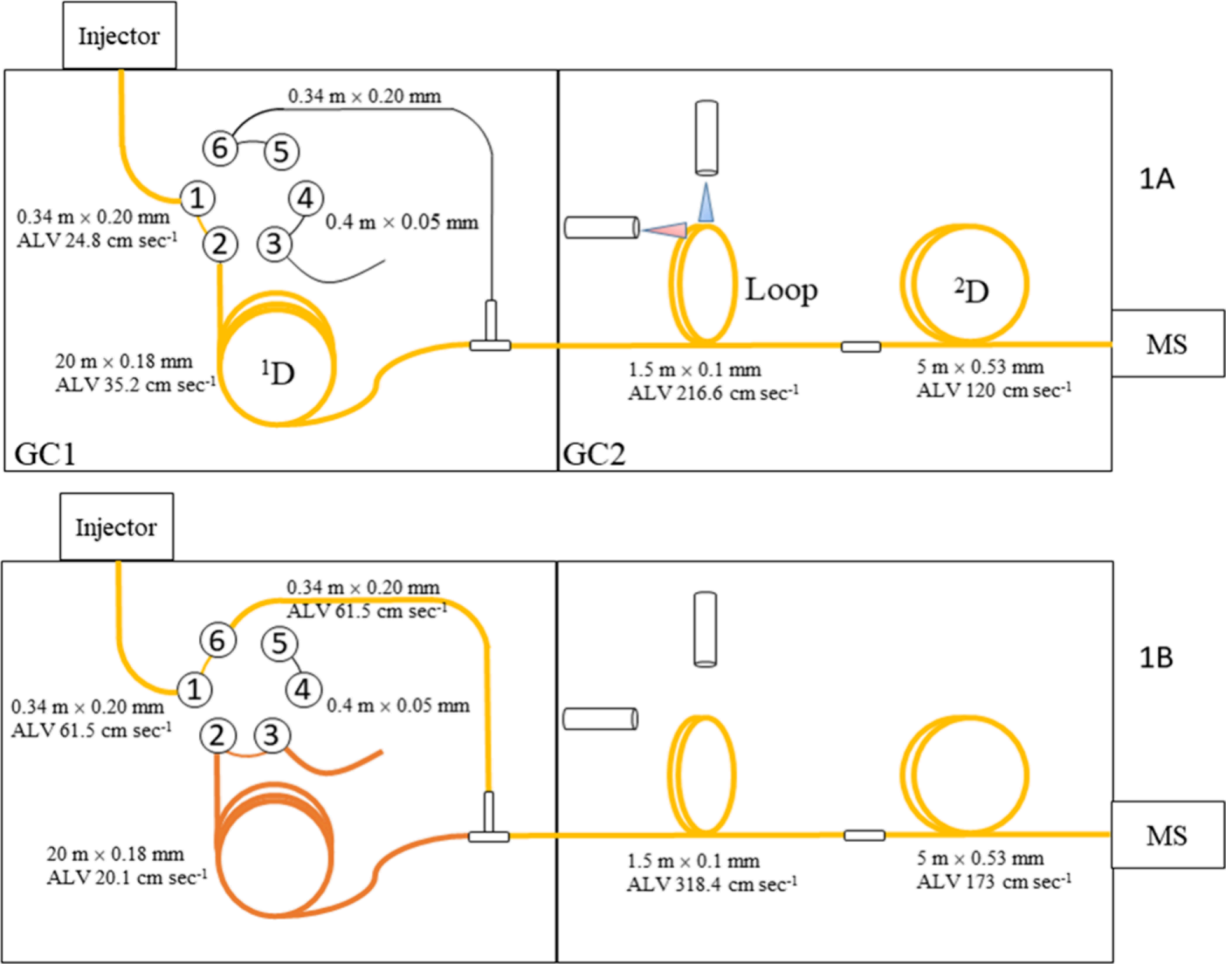
Schematic representation of the analytical system along with average linear velocities (ALV). The eGC×LP-GC-QMS configuration is reported in 1A, and the LP-GC-QMS configuration is reported in 1B.

Specifically in Figure 1A is reported the eGC×LP-GC-QMS configuration and in Figure 1B the LP-GC-QMS configuration also allowing the backflush of the ^1^D enantio-column.

The eGC×LP-GC-QMS (Figure 1A) analyses were performed by injecting 2 μL (splitless mode) with the following oven temperature program: 80 °C (2 min) to 120 °C at 10 °C min^–1^, increased to 230 °C at 2 °C min^–1^ (30 min), with a total run time of 91 min (after 71 min the valve changes position to allow the ^1^D backflush for 20 min). A positive offset of 10 °C was employed in the second oven.

The LP-GC-QMS (Figure 1B) analyses were carried out by injecting 4 μL (splitless mode), applying the following temperature program to the oven GC2 where is placed the wide-bore column: 30 °C (3 min), increased at a rate of 30 °C min^–1^ to 320 °C. The temperature of the GC1 was raised (30 °C min^–1^) starting from 30 °C and kept at 230 (maximum operating temperature of the ^1^D enantio-column) until the end of the chromatographic run. Total analysis time was 12.6 min (last eluting compound at approximately 9.7 min). During the LP-GC-QMS analysis, about 15% of the column flow is directed to the exhaust. Helium (99.999%) was used as carrier gas; detailed information regarding flows and average linear velocities (ALV) is reported in results and discussion paragraphs. Regarding the mass spectrometer, electron ionization was performed at 70 eV, and the interface and ion source temperatures were both at 230 °C.

To perform targeted and potentially untargeted analysis, both SCAN and selected ion monitoring (SIM) mode were performed with both system configurations. Acquisition frequencies of 14 and 33 Hz were set for SCAN and SIM modes respectively, allowing to obtain good peak reconstruction with approximately 15 data points per peak for the eGC×LP-GC-QMS (in SIM mode). The peak capacity obtained in the ^2^D is slightly lower than that achievable with columns having an ID of 0.1–0.25 mm.^1^ While during the LP-GC-QMS runs the acquisition frequencies were 3 and 10 Hz, for SCAN and SIM mode, respectively. The SCAN acquisition was performed in the following range: 40–400 *m*/*z* while for the SIM the three most abundant fragments of each pesticide were selected (the most abundant one as quantifier and the other two as qualifiers). Identification of the targeted compounds was performed in SCAN mode by using the Pesticides 2.0 spectral database (Shimadzu). Selected ion fragments for SIM acquisition along with apple maximum residual limits (MRLs), and chiral center information, are given in Table S1.

## Results and Discussion

### Consideration of the Chromatographic Setup

The goal of the present research was to develop an instrumental configuration that allows for an easy switch between eGC×LP-GC-QMS and LP-GC-QMS, in order to exploit the advantages of both techniques. By employing a slightly more complex system, we were able to enhance the chromatographic resolution, improve the analyte detectability, and expand the range of compounds that can be analyzed. To the best of the authors’ knowledge, this is the first description of such type of approach.

To achieve such a scope, a 6 ports-two positions valve was installed in the first GC oven.

Enantiomeric resolution requires the use of a chiral column, however, a major limitation of these stationary phases is their low maximum operating temperature, which hinder the elution of high-boiling compounds.^7^ To address this issue, the backflush technique was applied to the ^1^D enantio-column, enabling the removal of high-boiling compounds and keeping the column clean. The innovative idea of this work is also to use this instrumental setup to perform LP-GC-QMS analyses, which, despite part of the flow (about 15%) being directed to the exhaust, allows to reach a satisfactory sensitivity.

Proper operation of both setups required careful optimization of column dimensions and carrier gas flow conditions.

In detail, for the eGC×LP-GC-QMS configuration the following column setup of 20 m × 0.18 mm I.D. + 1.5 m × 0.10 mm I.D. + 5 m × 0.53 mm ID was employed, generating an equivalent column of 35.9 m × 0.18 mm I.D. that was set in the GC software (the calculations of equivalent columns and flow rates were performed as reported by Tranchida et al.).^20^ An inlet pressure of 364.5 kPa (working under constant ALV mode) was applied at the beginning of the analysis, obtaining a column flow of 1.8 mL min^–1^. In such conditions ALVs of 35.2, 216.6, and 120.0 cm sec^–1^ were obtained in the ^1^D, loop and ^2^D, respectively. The ^2^D dimension separation was performed under vacuum conditions (−81.2 kPa at the beginning of the analysis) using a short, megabore column (5 m × 0.53 mm I.D.) achieving fast separation conditions.

For the backflush procedure, after the target molecule elution (71 min), the position valve was switched (Figure 1B). During the backflush the oven temperature was kept at 230 °C for 20 min and the injector pressure was increased up to 750 kPa (the inlet pressure was approximately 490 kPa at the end of the separation time) to facilitate the column cleaning.

Considering the ^1^D column (20 m × 0.18 mm I.D.) and restrictor capillary dimensions (0.4 m × 0.05 mm I.D.), an equivalent column of 90 m × 0.18 mm I.D. was used for the following calculations. In such conditions, 15% of the flow was diverted to the ^1^D column and expelled from the capillary connected in position 3 of the valve. The capillary was connected to a lab fume hood at ambient pressure to extract the vapors.

With an average linear velocity of 24.4 cm sec^–1^ (hold up time approximately 370 s), the ^1^D column was flushed three times during the backflush. In the meantime, a flow of 7.0 mL min^–1^ (compatible with the MS vacuum pump extraction capacity) was diverted to the wide-bore column. After the analyses, a blank run, injecting only solvent, was acquired to assess the absence of carryover of the system.

To demonstrate the capabilities of the developed system, Figure 2 presents the chromatogram of the 56 pesticides (at a concentration of 33 mg L^–1^) acquired in SCAN mode. The separated enantiomers are highlighted with circles, and the corresponding identification numbers are provided in Table 1. During the LP-GC-QMS analysis, the switching valve was in the same position as used during the backflush step (equivalent column dimensions 15.94 m × 0.18 mm I.D.).

**Figure 2:**
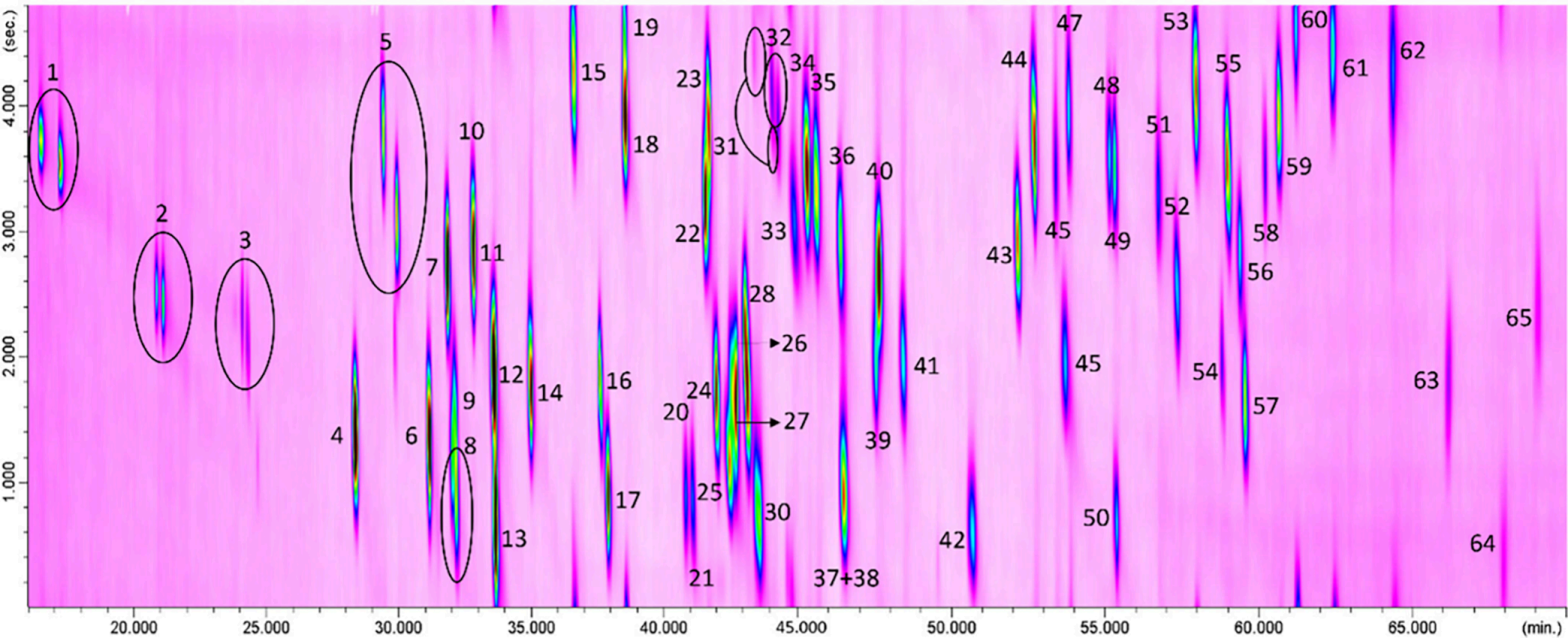
eGC×LP-GC-QMS bidimensional chromatogram acquired in SCAN mode of the 56 pesticides with the 7 separate enantiomers circled.

**Table 1:** List of Analyzed Pesticides in Solvent Analyzed by eGC×LP-GC-QMS^a^

No.	Compound	t_R_ (min)	s	n	s/n	Concn (μg L^–1^)	Absolute injected amount (pg)
1	Methamidophos (A)	16.66	200	20	10	80	72
	Methamidophos (B)	17.32	340	25	14	80	88
2	Acephate (A)	20.9	7534	40	188	3000	3420
	Acephate (B)	21.14	7000	56	125	3000	2580
3	Dibrom (A)	24.14	280	17	16	40	40
	Dibrom (B)	24.3	200	10	20	40	40
4	Fonofos	28.38	170	17	10	10	20
5	Propetamphos (A)	29.41	135	10	14	80	80
	Propetamphos (B)	29.98	250	20	13	80	80
6	Spiroxamine I	31.12	240	25	10	10	10
7	Hydroprene	31.9	300	17	18	40	80
8	Benoxacor (A)	32.04	346	17	20	165	148.5
	Benoxacor (B)	32.2	626	17	37	165	181.5
9	Heptachlor	32.12	345	23	15	40	80
10	Acetochlor	32.81	317	23	14	80	160
11	Vinclozolin	33.55	450	45	10	300	600
12	Spiroxamine II	33.61	320	30	11	10	10
13	Fenpropidin	33.63	370	35	11	10	20
14	Metalaxil	34.96	330	22	15	40	80
15	Metolachlor	36.67	280	20	14	20	40
16	Kinoprene	37.62	190	20	10	165	330
17	Malathion	37.94	400	25	16	80	160
18	Ethofumesate	38.58	240	25	10	40	80
19	Butralin	38.6	351	18	20	40	80
20	Fostiazate I	40.86	170	17	10	165	165
21	Fostiazate II	41.11	180	18	10	165	165
22	Isofenphos	41.65	950	75	13	40	800
23	Dinobuton	41.74	185	18	10	80	160
24	Amiprofos-methyl	41.96	450	45	10	80	160
25	Carbetamide	42.45	790	50	16	80	160
26	Beflubutamid	42.55	570	50	11	40	80
27	Phenthoate	42.71	800	55	15	40	80
28	Procimidone	43.05	320	25	13	20	40
29	Furalaxyl	43.12	690	40	17	20	40
30	Triadimenol	43.61	240	20	12	40	80
31	Chlorflurenol (A)	44.00	250	25	10	300	300
	Chlorflurenol (B)	44.24	240	20	12	300	300
32	Fipronil (A)	44.09	265	13	20	165	165
	Fipronil (B)	44.32	320	13	25	165	165
33	Mephosfolan	44.82	447	33	14	300	600
34	Napropamide	45.32	270	20	14	20	40
35	Hexaconazole I	45.65	480	50	10	80	80
36	Profenofos	46.4	490	40	12	80	160
37	Hexaconazole II	46.53	520	50	10	80	80
38	Flutriafol	46.53	800	60	13	40	80
39	Iprovalicarb I	47.55	360	25	14	80	80
40	Flamprop-methyl	47.75	525	50	11	10	20
41	Iprovalicarb II	48.46	400	30	13	80	80
42	Ancymidol	50.69	120	10	12	40	80
43	Sulprofos	52.23	290	20	15	40	80
44	Benalaxyl	52.75	210	20	11	10	20
45	Propiconazole I	53.5	280	20	14	80	80
46	Ofurace	53.79	260	15	17	80	160
47	Propiconazole II	53.91	250	20	13	80	80
48	Propargite I	55.24	180	15	12	20	20
49	Propargite II	55.41	210	15	14	20	20
50	Clodinafop-propargyl	55.45	90	9	10	80	160
51	Epoxiconazole	56.82	200	15	13	165	330
52	Mefenpyr-diethyl	57.4	650	20	33	20	40
53	Bifenthrin	58.09	260	20	13	10	20
54	Tetramethrin I	58.88	405	40	10	40	16.8
55	Fenpropathrin	59.07	920	70	13	40	80
56	Methoxychlor	59.48	390	40	10	80	160
57	Tetramethrin II	59.62	655	40	16	20	31.6
58	Phenothrin II	60.32	200	20	10	10	15
59	Phenothrin I	60.75	230	20	12	40	20
60	Leptophos	61.35	365	30	12	40	80
61	Pyriproxyfen	62.59	380	20	19	40	80
62	Fenarimol	64.50	410	35	12	80	160
63	Dialifos	66.29	200	20	10	40	80
64	Permethrin I	68.17	300	15	20	40	40
65	Permethrin II	69.21	280	15	19	40	40

a Separate chiral compounds are reported twice. t_R_, retention time; s, signal; n, noise; concn, concentration of the injected solution employed for the calculation.

The initial injector pressure was 308.5 kPa, generating an average linear velocity of 173.0 cm sec^–1^ inside the analytical column (the wide-bore column 5 m × 0.53 mm I.D. × 0.53 μm *d*_*f*_). While into the uncoated segments the ALVs were equal to 61.5 and 318.4 cm sec^–1^ respectively. A negative pressure of −75.1 kPa in the inlet of the wide-bore column was generated at the beginning of the analysis. During the LP-GC-QMS analysis, 86% of the flow was directed into the 0.53 mm I.D. column while the remaining was directed/discharged by the third branch connected to the Y union. To demonstrate the capabilities of the LP-GC-QMS configuration, Figure 3 shows the chromatogram of the 56 pesticides standard (at a concentration of 3 mg L^–1^) acquired in SIM mode. All of the experiments in both eGC×LP-GC-QMS and LP-GC-QMS configurations were conducted in constant ALV mode.

**Figure 3:**
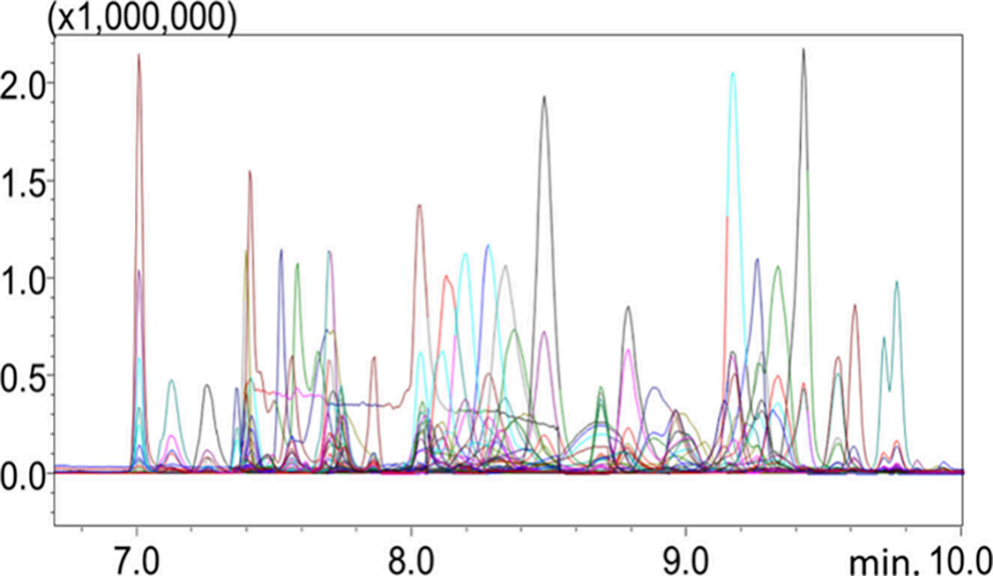
LP-GC-QMS chromatogram acquired in SIM mode for the 56 pesticides.

### Consideration of Chiral Pesticides

A significant portion of pesticides on the market have chiral properties with one or more stereocenters (typically C, N, or S atoms). The current regulation on maximum residue limits for pesticides is clear about isomers, indicating that all isomers of a pesticide must be included in the total residue assessment. However, the European Commission does not explicitly express about enantiomers, except for indoxacarb and benthiavalicarb.^21^

In the case of pesticides, chirality plays an important role. Some chiral pesticides can exhibit different activity against parasites, have a different biodegradation or can be toxic to human in one form but not the other.^22,23^ Given the stereoselectivity of chiral pesticides, the use of only the most active stereoisomer would certainly make pesticide use safer, reducing toxicological and environmental risks. Due to the limitations of synthesizing and/or isolating single enantiomers, most chiral pesticides are still sold as racemic mixtures.^24^ However, enantiomeric separation is necessary in correctly assessing the potential health risk of consuming contaminated food.

Among the seven chiral pesticides separated, acephate, methamidophos, dibrom (also known as naled), and propetamphos are organophosphates, fipronil is a phenylpyrazole, benoxacor is a benzoaxine, and chlorflurenol is a fluorenecarboxylic acid. Several articles in the literature have studied the enantioselectivity of chiral pesticides but more comprehensive research is required to fully understand their toxicological behavior to nontarget species.^25,26^

Emerick et al. investigated the toxicity of the two enantiomers of methamidophos both in vitro and vivo, in blood and brain samples from hens, human blood, and human cell culture samples. The work evaluated the ability of (+)-methamidophos and (−)-methamidophos to inhibit acetylcholinesterase and neuropathy target esterase. Based on in vitro results alone, the (+)-methamidophos enantiomer demonstrated a greater potential to induce delayed neuropathy than the (−)-methamidophos enantiomer in both humans and hens. However, discrepancies in toxicity were observed between the in vitro and in vivo results.^27^ In the case of fipronil, two studies have tested (R) and (S) enantiomers in species such as *Apis mellifera* (honeybee) and *Ceriodaphnia dubi*.^28,29^ In honeybees, contact and oral toxicities of the R-enantiomer, S-enantiomer, and racemate did not show statistically significant differences. In contrast, acute toxicity tests with *C. dubia* showed that S-fipronil was more toxic than both racemate and R-fipronil.

Regarding benoxacor, Sihong Liu et al. studied its chiral toxicity on zebrafish embryos, showing that racemic-benoxacor had higher toxicity than the others, with R-benoxacor being the least toxic.^30^

### eGC×LP-GC-QMS and LP-GC-QMS Performance

Initially, a mixture of saturated alkanes (C_7_–C_30_) was injected to investigate the range of analyzable compounds using the eGC×LP-GC-QMS system. Under the applied analytical conditions, it was possible to confirm the elution of C_26_ alkane at 224 °C (during the temperature program), confirming the suitability of the developed system for semivolatile analytes. The chromatogram acquired by injecting the C_7_–C_30_ standards solution is reported in Figure S1.

Regarding the LP-GC-QMS configuration, some additional consideration must be done. To connect the injector to the wide-bore column, two uncoated capillary columns and the switching valve were installed in the first GC oven, which also houses the ^1^D chiral column (maximum operating temperature: 230 °C). A mixture of linear alkanes (C_7_–C_40_) was injected to evaluate (i) the impact of the chiral column’s thermal limitation on the injection of high-boiling compounds and (ii) the volatility range amenable using the LP-GC-QMS configuration. Regarding the first point, results demonstrated that the uncoated columns and the switching valve, employed as transfer lines within the first oven, did not hinder the elution of compounds with boiling points exceeding 230 °C (up to equivalent to C_40_). As for the second point, the analysis revealed a broad operational range with C_40_ eluting at 295 °C (12.48 min) during the temperature program. Additionally, the full width at half-maximum (fwhm) of selected alkanes, namely, C_12_, C_25_, and C_40_ was assessed, yielding values of 1.5, 1.9, and 2.9 s, respectively, with an average fwhm of 2.1 s. The chromatogram of the C_7_–C_40_ mixture is reported in Figure S2.

To evaluate the capability of the eGC×LP-GC-QMS method to fulfill the MRLs reported for apple samples, matrix-matched calibration curves (*n* = 3) were constructed by spiking a blank apple sample (free of pesticide residues). It is noteworthy that the sample preparation method simply involved an extraction step. The lowest point of the calibration range was equal at least to the MRL of each pesticide and corresponded to the concentration of the single compound in a spiked sample generating a signal-to-noise (*s/n)* ≥ 10, except for fipronil and metalaxyl (MRL 5 and 10 μg kg^–1^ respectively) with LOQ values of 10 and 50 μg kg^–1^. LOQs were calculated using the s/n values of the highest and most abundant modulations of each compound. Values were reported in Table S1. Thanks to the properties of the employed chiral column, enantiomeric separation was achieved for seven pesticides (acephate, benoxacor, chlorflurenol, dibrom, fipronil, methamidophos, and propetamphos). While further studies are certainly necessary to broaden the scope of enantiomeric separation, the results obtained represent a significant step forward in the analysis of chiral pesticides.

Moreover, it is unclear whether the failure to achieve separation for the remaining pesticides may be attributed to either limitations of the chiral column or the composition of the standard mixtures. In fact, for each pesticide, the presence of both enantiomers or, if present, the specific enantiomeric form was not explicitly indicated on the label.

Furthermore, to compare the analytes, detectability of the two proposed approaches was compared by injecting increasingly diluted standard solutions and calculating the s/n ratio. Specifically, the comparison was performed using the s/n value closest to 10. For the eGC×LP-GC-QMS method, the most intense modulation was used. The obtained s/n values, along with the corresponding concentrations and absolute amounts injected onto the column, are reported in Tables 1 and 2.

**Table 2:** List of Analyzed Pesticides in Solvent Analyzed by LP-GC-QMS^b^

Compound	t_R_ (min)	s	n	s/n	Concn (μg L^–1^)	Abs injected amount (pg)
Methamidophos	4.99	1340	60	22	80	320
Dibrom	4.99	477	30	16	20	80
Acephate	5.92	9120	360	25	3000	12000
Fonofos	7.02	380	30	13	2.5	10
Propetamphos	7.13	980	90	11	2.5	10
Benoxacor	7.25	870	40	22	40	160
Heptachlor	7.38	480	20	24	20	80
Hydroprene	7.41	1065	50	21	5	20
Acetochlor	7.42	470	48	10	5	20
Spiroxamine I	7.43	1500	70	21	10	20
Vinclozolin	7.46	1130	70	16	40	160
Fenpropidin	7.55	720	75	10	20	80
Metalaxil	7.55	6935	400	17	10	40
Spiroxamine II	7.60	1330	70	19	10	20
Chlorflurenol	7.64	850	40	21	40	160
Ethofumesate	7.69	880	65	14	5	20
Metolachlor	7.70	1240	70	18	2.5	10
Malathion	7.74	1680	160	11	20	80
Kinoprene	7.76	670	40	17	20	80
Butralin	7.86	365	30	12	5	20
Fostiazate^a^	7.93	2100	50	42	20	80
Amiprofos-methyl	8.02	1750	80	22	20	80
Isofenphos	8.03	540	50	11	2.5	10
Phenthoate	8.03	650	60	11	5	20
Procimidone	8.09	3250	140	23	5	20
Dinobuton	8.10	8060	270	30	40	160
Furalaxyl	8.11	3650	200	18	2.5	10
Beflubutamid	8.16	1460	100	15	2.5	10
Triadimenol	8.16	1200	90	13	10	40
Mephosfolan	8.17	1040	100	10	40	160
Fipronil	8.23	424	21	20	20	80
Napropamide	8.26	820	40	21	2.5	10
Profenofos	8.27	7315	255	29	80	320
Carbetamide	8.29	2100	215	10	20	80
Hexaconazole^a^	8.29	8520	300	28	10	40
Flutriafol	8.29	1785	140	13	5	20
Iprovalicarb I	8.39	700	35	20	10	20
Flamprop-methyl	8.45	4000	225	18	2.5	10
Iprovalicarb II	8.46	640	35	18	10	20
Ancymidol	8.62	500	30	17	10	40
Sulprofos	8.67	1235	110	11	10	40
Benalaxyl	8.76	1650	90	18	5	20
Propiconazole^a^	8.85	625	60	10	10	40
Ofurace	8.94	970	75	13	10	40
Clodinafop-propargyl	8.94	320	30	11	10	40
Propargite^a^	8.94	1800	70	26	2.5	10
Epoxiconazole	9.09	825	35	24	20	80
Mefenpyr - diethyl	9.15	16200	170	95	2.5	10
Bifenthrin	9.16	3605	260	14	2.5	10
Methoxychlor	9.16	1540	130	12	20	80
Tetramethrin^a^	9.22	3640	190	19	5	20
Fenpropathrin	9.22	3430	365	9	10	40
Leptophos	9.29	2170	160	14	10	40
Phenothrin^a^	9.32	3525	150	24	5	20
Pyriproxyfen	9.39	2790	90	31	2.5	10
Fenarimol	9.49	910	75	12	10	40
Dialifos	9.49	680	55	12	10	40
Permethrin I	9.70	1285	120	11	10	20
Permethrin II	9.74	1860	120	16	10	20

a t_R_, retention time; s, signal; n, noise; concn, concentration of the injected solution employed for the calculation.

b Co-eluted isomers.

Using the eGC×LP-GC-QMS approach, s/n ratios ranged from approximately 10 for spiroxamine I and II, with an absolute injected amount of 10 pg, to 27 for acephate (a single enantiomer) with an absolute injected amount of 3.42 ng (values are reported in Table 1). For the LP-GC-QMS method, s/n ratios ranged from 95 for mefenpyr-diethyl at an absolute injected amount of 10 pg to 25 for acephate with an absolute injected amount of 12 ng (values are reported in Table 2).

In general a lower analytes detectability was obtained by using the LP-GC-QMS approach. Considering that the analytical column used in both approaches is the same, that the analytical conditions are similar (with a higher linear velocity under low-pressure conditions), and that the acquisition frequency is slightly higher in the two-dimensional approach, the observed differences can be attributed to the modulation process. In GC×GC, a compound should be modulated at least 3 times, resulting in the detector receiving a percentage of the total amount based on the number of modulations, despite the band compression effect.

### Benefit of eGC×LP-GC-QMS

The benefits of GC×GC are well-known and well established; GC×GC can be particularly valuable for the separation and quantification of target compounds in complex samples. In targeted analyses of complex matrices, coelutions accompanied by spectral interferences may occur, potentially compromising quantification accuracy.

In the present study, in addition to the enantiomeric separation achieved using eGC×LP-GC-QMS, particular attention was given to identifying and separating potential matrix-derived interferents. In several cases, the use of eGC×LP-GC-QMS enabled chromatographic separation in the second dimension of matrix coextracts that produced fragment ions potentially interfering with those of the target compounds.

For example, Figure 4 shows the raw chromatogram along with a two-dimensional expansion illustrating the second-dimension separation of hydropene characterized by ions with *m*/*z* 139, 111, and 81 from matrix-derived interferents also exhibiting a fragment with *m*/*z* 81. In a 1D analysis, this would result in coelution, altering the expected intensity ratios of the selected ions and potentially compromising compound identification.

**Figure 4:**
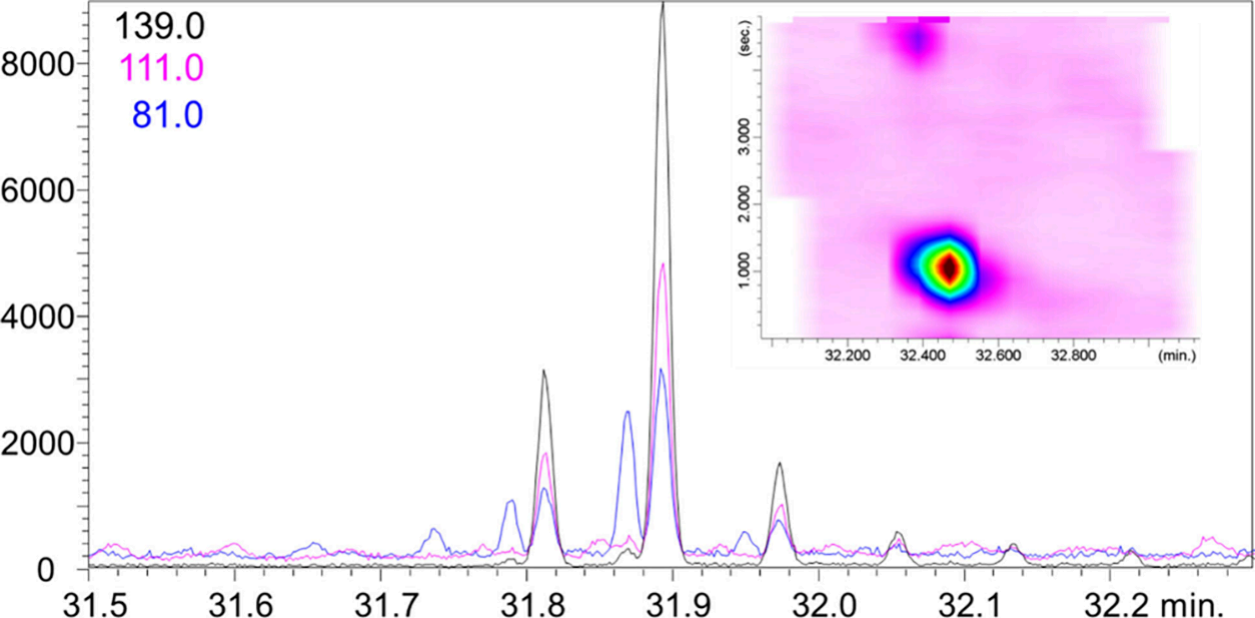
eGC×LP-GC-QMS RAW and 2D plot chromatograms highlighting the 2D separation between matrix interferent ion (*m*/*z* 81) and hydropene.

Another critical case arises when the interfering fragment coincides with the quantifier ion selected for a target compound. Figure 5 illustrates an example in which the quantifier ion (*m*/*z* 94) for the pesticide methamidophos is also present in coextracted matrix interferences.

**Figure 5:**
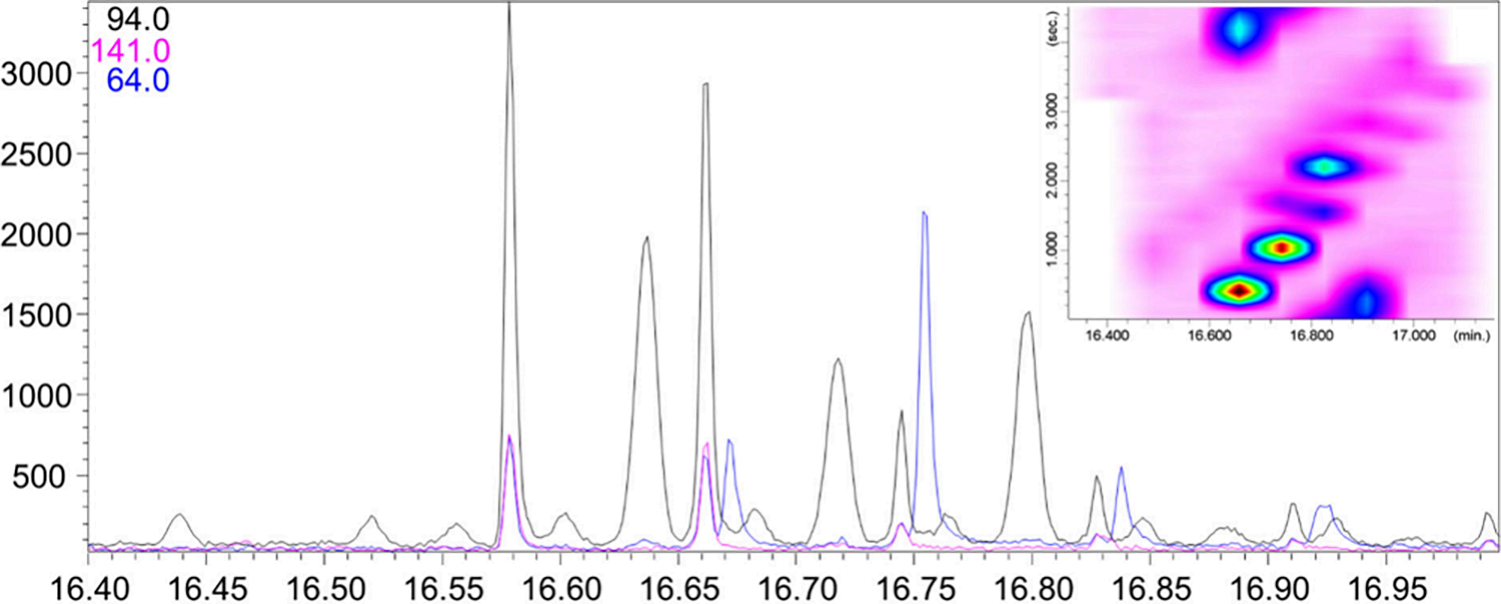
eGC×LP-GC-QMS RAW and 2D plot chromatograms highlighting the 2D separation between matrix interferent ion (*m*/*z* 94) and methamidophos.

In such instances, the automatic integration function of data analysis software may fail to recognize the target compound correctly. This can lead to inaccurate quantification, with most often an overestimation of the target analyte.

## Conclusions

This study presents a novel and flexible instrumental configuration that enables switching between eGC×LP-GC-QMS and LP-GC-QMS using a single analytical system. By incorporating a six-port switching valve, the developed system enables targeted chiral analysis, untargeted analysis, and rapid screening of pesticides in complex food matrices without the need for additional injectors or complex instrument modifications.

The backflush technique extends the lifetime of the chiral column by removing high-boiling matrix interferences, while the LP-GC-QMS mode enables faster separations, reducing analysis time and energy consumption; these benefits are achieved at the expense of chromatographic resolution.

The use of a wide-bore ^2^D column under LP conditions enhances sensitivity, peak shape, and robustness, particularly for thermally labile compounds. The dual-oven configuration, with the wide-bore column positioned in a separate oven, overcomes the temperature limitations of chiral stationary phases, broadening the range of analyzable compounds including high-boiling pesticides in food samples.

Validation studies demonstrated satisfactory figures of merit for both configurations. The eGC×LP-GC-QMS approach proved particularly valuable in resolving coeluting matrix interferences that could otherwise compromise quantification in monodimensional analyses.

Future work could explore the coupling of this system with advanced mass spectrometry techniques (e.g., tandem MS) for improved selectivity as well as its application to other complex matrices beyond apple samples. Overall, this study highlights the potential of flexible, multifunctional GC systems to address diverse analytical challenges in food safety and environmental monitoring.

## Supplementary Material



## Notes

Details about analyzed pesticides; eGC×LP-GC-QMS and LP-GC-QMS chromatograms (PDF)

## References

[1] Mondello L., Cordero C., Janssen H. G., Synovec R. E., Zoccali M., Tranchida P. Q. (2025). Comprehensive two-dimensional gas chromatography–mass
spectrometry. Nature Reviews Methods Primers..

[2] Devers J., Pattison D. I., Hansen A. B., Christensen J. H. (2025). Comprehensive
two-dimensional gas chromatography as a tool for targeted and non-targeted
analysis of contaminants of emerging concern in wastewater. Talanta..

[3] Kandula J. S., Rayala V. V. S. P. K., Pullapanthula R. (2023). Chirality:
An inescapable concept
for the pharmaceutical, biopharmaceutical, food, and cosmetic industries. Separation Science Plus..

[4] Alvarez-Rivera G., Bueno M., Ballesteros-Vivas D., Cifuentes A. (2020). Chiral analysis
in food science. TrAC Trends in Analytical Chemistry..

[5] Engel K. H. (2020). Chirality:
An important phenomenon regarding biosynthesis, perception, and authenticity
of flavor compounds. J. Agric. Food Chem..

[6] Galletta M., Zoccali M., Malegori C., Oliveri P., Tranchida P. Q., Mondello L., Mondello M. (2024). Flow-modulation
comprehensive two-dimensional
enantio-gas chromatography: A valid and flexible alternative to heart-cutting
multidimensional enantio-gas chromatography. Talanta..

[7] Betzenbichler G., Huber L., Kräh S., Morkos M.-L. K., Siegle A. F., Trapp O. (2022). Chiral stationary phases and applications in gas chromatography. Chirality..

[8] Menestrina F., Ronco N. R., Romero L. M., Castells C. B. (2018). Enantioseparation
of polar pesticides on chiral capillary columns based on permethyl-β-cyclodextrin
in matrices of different polarities. Microchemical
Journal..

[9] Critto E. F., Prince D. L., Lancioni C., Castells C. B. (2024). Enantio-separation
of pesticides by gas chromatography: Measurement of association constants
enantiomer–chiral selector. Journal of
Chromatography A.

[10] Meng Z., Cui J., Li R., Sun W., Bao X., Wang J., Zhou Z., Zhu W., Chen X. (2022). Systematic
evaluation
of chiral pesticides at the enantiomeric level: A new strategy for
the development of highly effective and less harmful pesticides. Science of The Total Environment..

[11] Gray B. P., Teale P. (2010). The use of a simple
back-flush technology to improve sample throughput
and system robustness in routine gas chromatography tandem mass spectrometry
analysis of doping control samples. Journal
of Chromatography A.

[12] Jacobs M. R., Gras R., Nesterenko P. N., Luong J., Shellie R. A. (2015). Back-flushing
and heart cut capillary gas chromatography using planar microfluidic
Deans’ switching for the separation of benzene and alkylbenzenes
in industrial samples. Journal of Chromatography
A.

[13] Edwards M., Górecki T. (2015). Inlet backflushing device for the improvement of comprehensive
two dimensional gas chromatographic separations. Journal of Chromatography A.

[14] de
Zeeuw J., Peene J., Jansen H. G., Lou X. W. (2000). A Simple
Way to Speed up Separations by GC-MS Using Short 0.53 mm Columns and
Vacuum Outlet Conditions. Journal of High Resolution
Chromatography.

[15] Ferracane A., Zoccali M., Arena A., Mondello M., Tran-chida P. Q., Mondello L. (2023). A dilute-and-inject
low-pressure gas chromatography-tandem
mass spectrometry method for phthalate determination in extra virgin
olive oil. Journal of Separation Science..

[16] Sapozhnikova Y., Lehotay S. J. (2015). Review of recent
developments and applications in low-pressure
(vacuum outlet) gas chromatography. Anal. Chim.
Acta.

[17] Tranchida P. Q., Franchina F. A., Dugo P., Mondello L. (2014). Flow-modulation low-pressure
comprehensive two-dimensional gas chromatography. Journal of Chromatography A.

[18] Corbally M. A., Hinz N. S., Freye C. E. (2023). Comprehensive two-dimensional
gas
chromatography under low-pressure conditions. Journal of Chromatography A.

[19] European Committee for Standardization (CEN) . EN 15662: Food of plant origin — Multimethod for the determination of pesticide residues using GC- and LC-based analysis following ace-tonitrile extraction/partitioning and clean-up by dispersive SPE — Modular QuEChERS method, 2018. www.cen.eu.

[20] Tranchida P. Q., Zoccali M., Franchina F. A., Cotroneo A., Dugo P., Mondello L. (2013). Gas velocity at the
point of re-injection: An additional
parameter in comprehensive two-dimensional gas chromatography optimization. Journal of Chromatography A.

[21] Regulation (EC) No. 396/2005 of the European Parliament and of the Council of 23 February 2005 on maximum residue levels of pesticides in or on food and feed of plant and animal origin and amending Council Directive 91/414/EEC; European Union, 2005.

[22] Wong C. S. (2006). Environmental
fate processes and bio-chemical transformations of chiral emerging
organic pollutants. Analytical and Bioanalytical
Chemistry..

[23] de
Albuquerque N. C. P., Carrao D. B., Habenschus M. D., de Oliveira A. R. M. (2018). Metabolism studies of chiral pesticides: A critical
review. Journal of Pharmaceutical and Biomedical
Analysis..

[24] Carrao D. B., Perovani I. S., de Albuquerque N. C.
P., de Oliveira A. R. M. (2020). Enantioseparation
of pesticides: A critical review. TrAC Trends
in Analytical Chemistry..

[25] Vashistha V. K., Sethi S., Mittal A., Das D. K., Pullabhotla R. V. S. R., Bala R., Yadav S. (2024). Stereoselective analysis
of chiral
pesticides: A review. Environmental Monitoring
and Assessment..

[26] Ji C., Song Z., Tian Z., Feng Z., Fan L., Shou C., Zhao M. (2023). Enantioselectivity
in the toxicological
effects of chiral pesticides: A review. Sci.
Total Environ..

[27] Emerick G. L., DeOliveira G. H., Oliveira R. V., Ehrich M. (2012). Comparative in vitro
study of the inhibition of human and hen esterases by methamidophos
enantiomers. Toxicology..

[28] Li X., Bao C., Yang D., Zheng M., Li X., Tao S. (2010). Toxicities
of fipronil enantiomers to the honeybee Apis mellifera L. and enantiomeric
compositions of fipronil in honey plant flowers. Environ. Toxicol. Chem..

[29] Wilson W. A., Konwick B. J., Garrison A. W., Avants J. K., Black M. C. (2008). Enantioselective
chronic toxicity of fipronil to Ceriodaphnia dubia. Arch. Environ. Contam. Toxicol..

[30] Liu S., Deng X., Bai L. (2021). Developmental
toxicity and transcriptome
analysis of zebrafish (Danio rerio) embryos following exposure to
chiral herbicide safener benoxacor. Sci. Total
Environ..

