# Supplementary material for: Integrated Analytical System for Comprehensive Two-Dimensional Enantio-Gas Chromatography and Low-Pressure Gas Chromatography Mass Spectrometry Utilizing a Switching Valve: Design and Optimization
Source: Anal Chem. 2025 Nov 12;97(46):25406–15. doi: 10.1021/acs.analchem.5c05852 (PMC12658858; doi:10.1021/acs.analchem.5c05852)
Supplement: Supplementary file 1 [file ac5c05852_si_001.pdf]

Supporting information

Integrated analytical system for comprehensive two-dimensional enantio-gas chromatography and low-pressure gas chromatography mass spectrometry utilizing a switching valve: Design and Optimization

Giorgia Rinaldi[1], Antonio Ferracane[1], Mariosimone Zoccali[2,*], Luigi Mondello[1,3]

[1]Messina Institute of Technology c/o Department of Chemical, Biological, Pharmaceutical and Environmental Sciences, former Veterinary School, University of Messina, Viale G. Palatucci snc 98168 – Messina, Italy.

[2]Department of Mathematical and Computer Science, Physical Sciences and Earth Sciences, University of Messina, Viale F. Stagno d'Alcontres 31, 98166, Messina, Italy.

[3]Chromaleont s.r.l., c/o Messina Institute of Technology c/o Department of Chemical, Biological, Pharmaceutical and Environmental Sciences, University of Messina, Viale G. Palatucci snc 98168 – Messina, Italy.

Table of contents:

Table S1. List of compounds along with apple maximum residue limits (µg Kg^{-1}), limit of quantification (LOQ) (µg Kg^{-1}) calculated by injecting a pre-spiked apple sample, selected ion monitored (SIM), chiral center, and number of chiral centers.

Figure S1. Not-modulated eGC×LP-GC-QMS chromatogram of C_7-C_{30} linear alkanes (100 mg L^{-1}).

Figure S2. LP-GC-QMS chromatogram of C_7-C_{40} linear alkanes (100 mg L^{-1}).

*corresponding Author (mzoccali@unime.it)

Table S1. List of compounds along with apple maximum residue limits (µg Kg^{-1}), limit of quantification (LOQ) (µg Kg^{-1}) calculated by injecting a pre-spiked apple sample, selected ion monitored (SIM), chiral center, and number of chiral centers.

Compound	MRLs	LOQ	SIM			Chiral center	n° of chiral center
			Quantifier ion	Qualifier ions			
Acephate (A)<br>Acephate (B)	10	2.9<br>4.2	42	94	136	P	1
Acetochlor	10	5	146	175	132	C	1
Amiprofos - methyl	10	10	258	110	152	P	1
Ancymidol	10	10	107	121	228	C	1
Beflubutamid	20	5	91	176	221	C	1
Benalaxyl	10	5	148	91	206	C	1
Benoxacor (A)<br>Benoxacor (B)	10	11<br>9	120	176	259	C	1
Bifenthrin	10	5	181	166	176	C	2
Butralin	10	10	266	224	250	N	2
Carbetamide	10	10	119	93	72	C	1
Chlorflurecol (A)<br>Chlorflurecol (B)	10	5<br>5	181	152	216	C	1
Clodinafop - propargyl	20	10	266	238	349	C	1
Dialifos	10	10	208	173	76	C	1
Dibrom (A)<br>Dibrom (B)	10	5<br>5	109	79	145	C	1
Dinobuton	10	10	43	211	163	C	1
Epoxiconazole	10	10	192	165	138	C	2
Ethofumesate	30	10	207	161	137	C	1
Fenarimol	100	50	107	139	111	C	1
Fenpropathrin	10	5	97	55	181	C	1
Fenpropidin	10	5	98	96	117	C	1
Fipronil	5	10	366	351	255	S	1
Flamprop - methyl	10	5	105	77	276	C	1
Flutriafol	400	10	123	83	164	C	1
Fonofos	10	5	109	137	246	P	1
Fostiazate I<br>Fostiazate II	10	10<br>10	195	97	126	P	1
Furalaxyl	10	5	95	242	152	C	1
Heptachlor	10	5	100	272	65	C	5
Hexaconazole I<br>Hexaconazole I	10	2.5<br>5	83	214	82	C	1
Hydroprene	10	5	139	111	81	C	1
Iprovalicarb I<br>Iprovalicarb II	10	5<br>5	116	134	72	C	1
Isofenphos	10	5	58	213	121	P	1

Kinoprene	10	10	149	79	105	C	1
Leptophos	10	10	171	376	155	P	1
Malathion	20	5	127	173	125	C	1
Mefenpyr - diethyl	10	5	253	299	227	C	1
Mephosfolan	10	10	140	196	106	C	1
Metalaxyl	10	50	45	59	105	C	1
Methamidophos (A)<br>Methamidophos (B)	10	11<br>9	94	141	64	P	1
Methoxychlor	10	10	227	212	238	C	1
Metolachlor	50	5	162	238	163	C	2
Napropamide	10	5	72	100	128	C	1
Ofurace	10	5	132	160	232	C	1
Permethrin I<br>Permethrin II	50	25<br>25	183	127	91	C	2
Phenothrin I<br>Phenothrin II	20	3.75<br>2.5	123	183	81	C	2
Phenthoate	10	10	93	274	121	C	1
Procimidone	10	10	96	67	283	C	2
Profenofos	10	10	97	43	139	P	1
Propargite I<br>Propargite II	10	10<br>10	135	173	150	C-S	2-1
Propetamphos (A)<br>Propetamphos (B)	10	5<br>5	138	194	236	P	1
Propiconazole I<br>Propiconazole II	10	10<br>10	69	173	259	C	2
Pyriproxyfen	50	5	136	96	78	C	1
Spiroxamine I<br>Spiroxamine II	10	5<br>5	100	58	72	C	1
Sulprofos	10	5	156	140	322	P	1
Tetramethrin I<br>Tetramethrin II	10	2<br>8	164	123	81	C	2
Triadimenol	10	10	112	168	128	C	2
Vinclozolin	10	5	54	124	178	C	1

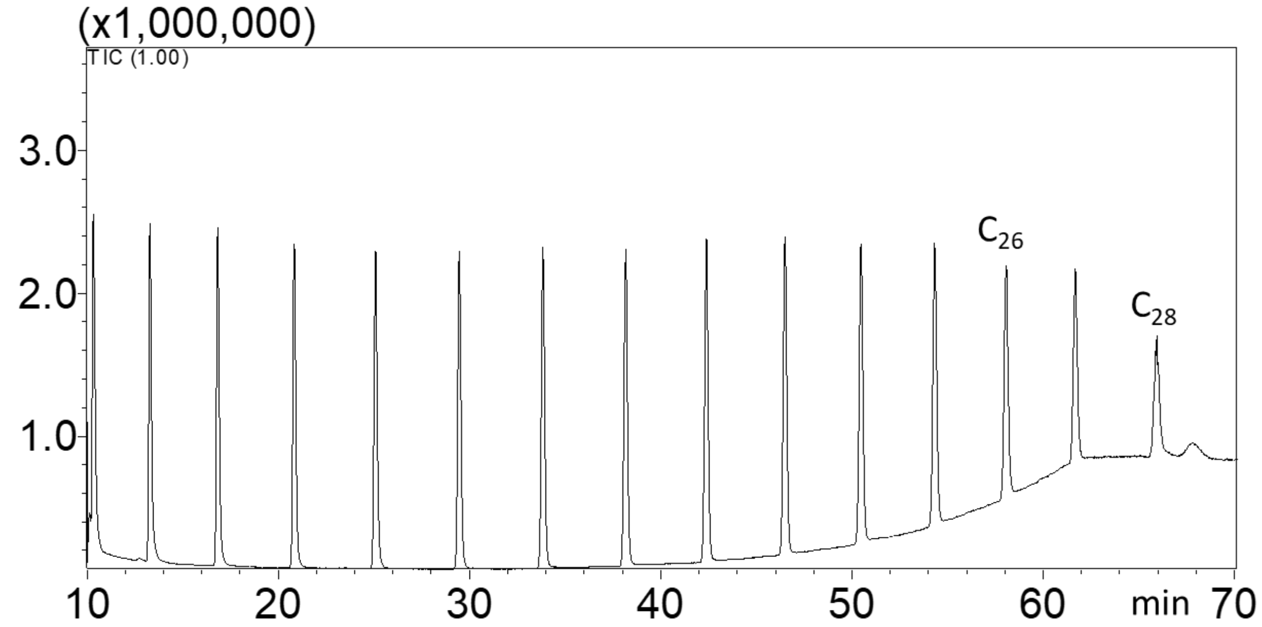

Figure S1. Not-modulated eGC×LP-GC-QMS chromatogram acquired in SCAN mode of C_7-C_{30} linear alkanes mixture (100 mg L^{-1}). Given the chromatographic and mass spectrometry acquisition conditions (see experimental conditions), alkanes in the range of C_{14} to C_{28} were detected.

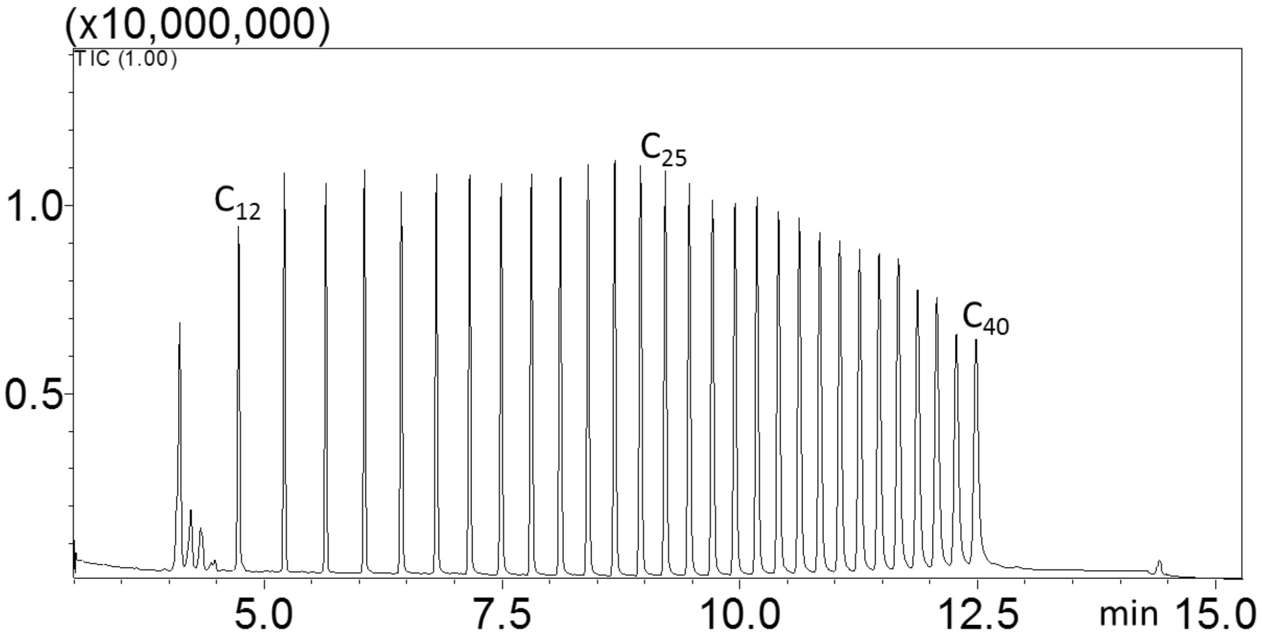

Figure S2. LP-GC-QMS chromatogram acquired in SCAN mode of C_7-C_{40} linear alkanes mixture (100 mg L^{-1}). Given the chromatographic and mass spectrometry acquisition conditions (see experimental conditions), alkanes in the range of C_{11} to C_{40} were detected.
